# Microarray Analysis of the Ler Regulon in Enteropathogenic and Enterohaemorrhagic *Escherichia coli* Strains

**DOI:** 10.1371/journal.pone.0080160

**Published:** 2014-01-14

**Authors:** Lewis E. H. Bingle, Chrystala Constantinidou, Robert K. Shaw, Md. Shahidul Islam, Mala Patel, Lori A. S. Snyder, David J. Lee, Charles W. Penn, Stephen J. W. Busby, Mark J. Pallen

**Affiliations:** School of Biosciences, University of Birmingham, Edgbaston, Birmingham, United Kingdom; University of Maryland School of Medicine, United States of America

## Abstract

The type III protein secretion system is an important pathogenicity factor of enteropathogenic and enterohaemorrhagic *Escherichia coli* pathotypes. The genes encoding this apparatus are located on a pathogenicity island (the locus of enterocyte effacement) and are transcriptionally activated by the master regulator Ler. In each pathotype Ler is also known to regulate genes located elsewhere on the chromosome, but the full extent of the Ler regulon is unclear, especially for enteropathogenic *E. coli*. The Ler regulon was defined for two strains of *E. coli*: E2348/69 (enteropathogenic) and EDL933 (enterohaemorrhagic) in mid and late log phases of growth by DNA microarray analysis of the transcriptomes of wild-type and *ler* mutant versions of each strain. In both strains the Ler regulon is focused on the locus of enterocyte effacement – all major transcriptional units of which are activated by Ler, with the sole exception of the *LEE1* operon during mid-log phase growth in E2348/69. However, the Ler regulon does extend more widely and also includes unlinked pathogenicity genes: in E2348/69 more than 50 genes outside of this locus were regulated, including a number of known or potential pathogenicity determinants; in EDL933 only 4 extra-LEE genes, again including known pathogenicity factors, were activated. In E2348/69, where the Ler regulon is clearly growth phase dependent, a number of genes including the plasmid-encoded regulator operon *perABC*, were found to be negatively regulated by Ler. Negative regulation by Ler of PerC, itself a positive regulator of the *ler* promoter, suggests a negative feedback loop involving these proteins.

## Introduction

Enteropathogenic (EPEC) and enterohaemorrhagic (EHEC) *Escherichia coli* are two pathotypes of this important gastrointestinal bacterium that can cause serious diarrhoeal disease in humans [1]. Many EHEC and EPEC strains possess a type III secretion system (T3SS) encoded by a pathogenicity island called the locus of enterocyte effacement (LEE) that is also found in the related bacterium *Citrobacter rodentium*, a mouse pathogen that is widely used as a model for the EHEC and EPEC strains [2]. Pathogenicity factors encoded within the LEE, specifically the type III secretion system and secreted effector proteins, are responsible for formation of the attaching and effacing (AE) lesion on the gut epithelium that is characteristic of these strains and required for intimate attachment of the bacteria [3]. The 41 genes of the LEE are arranged in 5 major polycistronic operons called *LEE1-5* along with a number of smaller transcriptional units [4]. Attaching and effacing pathogens, including EPEC strains such as E2348/69, O157:H7 EHEC strains and non-0157 EHEC strains, have distinct evolutionary histories but carry an overlapping core repertoire of pathogenicity genes, including the LEE and many effector genes outside the LEE, that have been acquired via horizontal gene transfer [5], [6], [7]. However, there are significant differences in overall pathogenicity between EHEC and EPEC strains, for example EHEC strains cause a more severe bloody diarrheal disease (haemorrhagic colitis) that is often accompanied by the life threatening complication, haemolytic uraemic syndrome (HUS) [8]. Such differences are presumably mainly determined by the differing contributions of the extra-LEE factors. Examples include differing arrays of T3SS effector proteins and the fact that the EHEC genome encodes a Shiga-like toxin responsible for serious pathology in the human host, while EPEC does not [8].

In addition to variation in the genomic arsenal of determinants, appropriate control of gene transcription may be critical in optimising pathogenicity [9], [10]. Type III secretion systems (T3SS) are generally acquired through horizontal gene transfer and therefore should employ a means of regulation that is easily integrated into the existing regulatory networks of the cell [11]. One way to achieve this integration is to have T3SS gene expression under the control of a master regulator, which multiple environmental signaling pathways can feed into. The master regulator for the LEE is the Ler protein, encoded by the first gene in the *LEE1* operon [12]. Ler is a transcriptional activator of the LEE: a homologue and also an antagonist of the genome organizer and silencer H-NS [13]. In addition to its H-NS-dependent role in activating most promoters of the LEE, Ler can activate the *LEE5* promoter in an H-NS-independent manner (reviewed in [14]). Ler has also previously been shown to act as a specific autorepressor of the *LEE1* promoter [15] while the LEE encoded regulator GrlA and the plasmid encoded regulator PerC (EPEC), or its EHEC homologues PchABC, have been shown to specifically activate *ler* transcription [13], [16], [17], [18], [19]. LEE gene expression is responsive to population status, via the AI-3 quorum sensing system activating the LysR type regulator QseA which in turn activates *LEE1* (*ler*) transcription [20], [21], [22]. Expression of the LEE is also known to be responsive to many environmental factors (reviewed in [23], [24]). One example is temperature: transcription of the LEE is up-regulated at 37°C and repressed (by H-NS) at 27°C [25]. Expression of the LEE is also dependent on the physiological state of the cell, for example growth phase. In glucose MOPS minimal medium, gene expression as assessed by microarray transcriptomics is maximal in late exponential phase and down-regulated during the transition to stationary phase [26]. Under some other growth conditions (LB broth) expression from LEE promoters, measured via transcription of a *lacZ* reporter gene, seemed to increase during the transition to stationary phase [20], [27].

Extra-LEE genes that are known to be members of the Ler regulon in EPEC include *espC*, encoding an autotransporter (Type V) extracellular serine protease, that is thought to play various roles in pathogenicity [28], [29], [30], [31]. The *espC* gene has previously been shown to be strongly activated by Ler, however in contrast the EHEC homologue of this gene *espP* was not found to be Ler-regulated [12]. Extra-LEE members of the Ler regulon in EHEC include *stcE*, a pO157–borne gene encoding a metalloprotease that is involved in intimate adherence of bacterium to gut epithelium [32] and *nleA*, encoding a T3SS-secreted effector protein [33]. However some of the many extra-LEE T3SS effectors of EHEC were previously thought not to be regulated by Ler e.g. EspJ and TccP [6], [34]. In addition, expression of long polar fimbriae of EHEC has been found to be reciprocally regulated by H-NS repression and Ler antagonism [35].

Here we will characterise and compare the Ler regulons for EPEC strain E2348/69 and EHEC strain EDL933. The regulon for Ler has previously been loosely defined at the transcriptional level for the closely-related Sakai strain of EHEC [36] where Ler regulation was mostly found to be confined to horizontally-transferred DNA. The LEE is inserted at the same *selC* locus in both EDL933 and E2348/69 strains, the most parsimonious interpretation being a single insertion event in a common ancestral strain [37]. Any differences in the Ler regulon between these two strains, within or outside the LEE, will reflect divergent adaptation to subsequent changes in the genome, for example plasmid acquisition, and are of interest from a regulon evolution point of view.

## Results

We constructed two validated mutant strains of *E. coli*: LBEC1 (EDL933 Δ*ler*) and LBEC2 (E2348/69 Δ*ler*), grew cultures of WT and parental mutant strains under conditions known to be inducing for the LEE to two different growth phases (mid and late log phase), harvested RNA and used this to perform microarray analysis of the transcriptomes. Microarray data has been deposited with the GEO database (http://www.ncbi.nlm.nih.gov/geo) with accession code GSE38876.

### Enteropathogenic E. coli

In mid-log phase cells a total of 85 genes are transcriptionally regulated: 62 genes, at 14 different loci, are activated by Ler (table 1) while 23 genes, at 6 loci, are repressed by Ler (table 2). Of the activated genes 49 (79%) are carried on or directly adjacent to mobile genetic elements (MGEs: prophage, integrative element or plasmid), while 11 of the repressed genes (48%) are carried on MGEs. If one compares the genes that are activated and repressed, two repressed genes (E2348C_0084 and E2348C_2114) are potentially expressed from promoters that are immediately divergent from an activated promoter.

**Table 1:** EPEC genes activated 2-fold or more by Ler at mid-log phase (OD_600_ = 0.4).

Systematic Gene Name	Fold Activation	Common Gene Name	Gene Product	MGE
E2348C_0081	6	*leuO*	DNA-binding transcriptional activator	
E2348C_0153	4	*-*	predicted fimbrial protein	
E2348C_0523	9	*pagP*	palmitoyl transferase for Lipid A	
E2348C_0683	4	*-*	hypothetical protein	PP2
E2348C_0684	21	*-*	SfpA (systemic factor protein A)-like protein	PP2
E2348C_1040	2	*-*	T3SS secreted effector NleI/NleG homolog	PP4
E2348C_1442	13	*-*	T3SS secreted effector NleA/EspI homolog	PP6
E2348C_1444	2	*-*	T3SS secreted effector NleH homolog	PP6
E2348C_2076	4	*yedR*	hypothetical protein	
E2348C_2111	4	*-*	hypothetical protein	IE3
E2348C_2112	3	*-*	hypothetical protein	IE3
E2348C_2705	3	*-*	predicted glycosyl transferase	
E2348C_2915	22	*espC*	extracellular serine protease EspC	IE5
E2348C_2916	3	*-*	T3SS secreted effector EspG homolog	IE5
E2348C_2917	3	*-*	hypothetical protein	IE5
E2348C_2918	3	*-*	hypothetical protein	IE5
E2348C_2920	27	*-*	hypothetical protein	IE5
E2348C_3262	2	*-*	predicted acyl-CoA synthase	
E2348C_3839	5	*yiaY*	predicted Fe-containing alcohol dehydrogenase	
E2348C_3929	2	*yicJ*	predicted transporter	±LEE
E2348C_3930	9	*espF*	LEE-encoded effector EspF	LEE
E2348C_3931	52	*orf29*	component of T3SS, SsaH family	LEE
E2348C_3932	42	*escF*	T3SS structure protein EscF	LEE
E2348C_3933	45	*cesD2*	chaperone CesD2	LEE
E2348C_3934	18	*espB*	translocator EspB	LEE
E2348C_3935	39	*espD*	translocator EspD	LEE
E2348C_3936	42	*espA*	translocator EspA	LEE
E2348C_3937	36	*sepL*	secretion switching protein SepL	LEE
E2348C_3938	32	*escD*	T3SS structure protein EscD	LEE
E2348C_3939	58	*eae*	intimin Eae	LEE
E2348C_3940	26	*cesT*	chaperone CesT	LEE
E2348C_3941	34	*tir*	translocated intimin receptor Tir	LEE
E2348C_3942	44	*map*	LEE-encoded effector Map	LEE
E2348C_3943	22	*cesF*	chaperone CesF	LEE
E2348C_3944	21	*espH*	LEE-encoded effector EspH	LEE
E2348C_3945	26	*sepQ*	T3SS structure protein SepQ	LEE
E2348C_3946	21	*orf16*	hypothetical protein	LEE
E2348C_3947	16	*orf15*	hypothetical protein	LEE
E2348C_3948	11	*escN*	translocator EscN	LEE
E2348C_3949	17	*escV*	translocator EscV	LEE
E2348C_3950	12	*mpc*	regulator Mpc	LEE
E2348C_3951	13	*espZ*	LEE-encoded effector EspZ	LEE
E2348C_3952	25	*rorf8*	chaperone of T3SS Rorf8	LEE
E2348C_3953	24	*escJ*	T3SS structure protein EscJ	LEE
E2348C_3954	28	*sepD*	secretion switching protein SepD	LEE
E2348C_3955	29	*escC*	T3SS structure protein EscC	LEE
E2348C_3956	23	*cesD*	chaperone CesD	LEE
E2348C_3957	14	*grlA*	positive regulator GrlA	LEE
E2348C_3958	7	*grlR*	negative regulator GrlR	LEE
E2348C_3959	9	*rorf3*	hypothetical protein	LEE
E2348C_3960	3	*escU*	T3SS structure protein EscU	LEE
E2348C_3968	21	*ler*	transcription regulator Ler	LEE
E2348C_3970	12	*espG*	LEE-encoded effector EspG	LEE
E2348C_3971	50	*rorf1*	hypothetical protein	LEE
E2348C_4274	2	*-*	predicted transporter	
E2348C_4348	2	*malF*	maltose transporter subunit	
E2348C_4349	2	*malE*	maltose transporter subunit	
E2348C_4350	3	*malK*	maltose transporter subunit	
E2348C_4351	3	*lamB*	maltose outer membrane porin (maltoporin)	
E2348C_4442	3	*eptA*	predicted metal dependent hydrolase EptA	
pMAR2_096	3	*-*	putative glutamate:gamma-aminobutyrate antiporter	pMAR2
pMAR2_097	2	*-*	putative glutamate racemase	pMAR2.

Fold activation shows expression in *ler* ^−+^/*ler* ^−^ cells. MGE, mobile genetic element; PP, prophage; IE, integrative element; LEE, locus of enterocyte effacement; ±LEE, directly adjacent to the LEE; pMAR2, plasmid. For each gene reported, *t*-test P-value was less than 0.05.

**Table 2:** EPEC genes repressed 2-fold or more by Ler at mid-log phase (OD_600_ = 0.4).

Systematic Gene Name	Fold Repression	Common Gene Name	Gene Product	MGE
E2348C_0084	2	*-*	fruR leader peptide	
E2348C_0318	2	*iraP*	hypothetical protein	
E2348C_1098	2	*-*	hypothetical protein	IE2
E2348C_1922	2	*yeaQ*	conserved inner membrane protein	
E2348C_2034	3	*sdiA*	DNA-binding transcriptional activator	
E2348C_2114	2	*-*	hypothetical protein	IE3
E2348C_3744	5	*chuT*	putative hemin binding protein	
E2348C_3745	6	*chuW*	putative coproporphyrinogen oxidase	
E2348C_3746	5	*chuX*	ShuX-like protein	
E2348C_3747	6	*chuY*	ShuY-like protein	
E2348C_3748	5	*chuU*	putative hemin permease	
E2348C_3749	6	*hmuV*	hemin importer ATP-binding subunit	
E2348C_3751	2	*hdeB*	acid-resistance protein	
E2348C_4667	3	*yjjZ*	hypothetical protein	
pMAR2_003	2	*bfpA*	major pilin structural unit bundlin	pMAR2
pMAR2_004	2	*bfpG*	lipoprotein	pMAR2
pMAR2_006	2	*bfpC*	predicted protein	pMAR2
pMAR2_007	2	*bfpU*	periplasmic protein	pMAR2
pMAR2_008	2	*bfpD*	nucleotide binding protein	pMAR2
pMAR2_020	3	*perA*	transcriptional activator of the bfp operon	pMAR2
pMAR2_021	2	*perB*	transcriptional regulator	pMAR2
pMAR2_022	4	*perC*	transcriptional regulator	pMAR2
pMAR2_036	2	*copB*	regulation of plasmid copy number	pMAR2

Fold repression shows expression in *ler* ^−^/*ler* ^+^ cells. MGE, mobile genetic element; IE, integrative element; pMAR2, plasmid. For each gene reported, *t*-test P-value was less than 0.05.

In late log phase cells 97 genes in total are regulated by Ler. Of these, 85 genes at 23 genetic locations are activated, of which 62 genes (73%) are carried on or directly adjacent to MGEs (table 3). Twelve genes are repressed by Ler, of which only 1 is adjacent to a MGE (table 4).

**Table 3:** EPEC genes activated 2-fold or more by Ler at late-log phase (OD_600_ = 0.9).

Systematic Gene Name	Fold Activation	Common Gene Name	Gene Product	MGE
E2348C_0081	6	*leuO*	DNA-binding transcriptional activator	
E2348C_0153	5	*-*	predicted fimbrial protein	
E2348C_0523	15	*pagP*	palmitoyl transferase for Lipid A	
E2348C_0683	3	*-*	hypothetical protein	PP2
E2348C_0684	35	*-*	SfpA (systemic factor protein A)-like protein	PP2
E2348C_0685	2	*-*	predicted late gene regulator	PP2
E2348C_0718	2	*-*	T3SS secreted effector NleH homolog	PP2
E2348C_0723	3	*-*	T3SS secreted effector EspJ homolog	PP2
E2348C_1040	2	*-*	T3SS secreted effector NleI/NleG homolog	PP4
E2348C_1041	2	*-*	T3SS effector-like protein NleB homolog	PP4
E2348C_1442	15	*-*	T3SS secreted effector NleA/EspI homolog	PP6
E2348C_1444	2	*-*	T3SS secreted effector NleH homolog	PP6
E2348C_1445	2	*-*	T3SS secreted effector NleF homolog	PP6
E2348C_1481	2	*yciW*	predicted oxidoreductase	
E2348C_2065	2	*rcsA*	DNA-binding transcriptional activator, co-regulator with RcsB	
E2348C_2076	6	*yedR*	hypothetical protein	
E2348C_2105	2	*-*	hypothetical protein	IE3
E2348C_2111	5	*-*	hypothetical protein	IE3
E2348C_2112	4	*-*	hypothetical protein	IE3
E2348C_2113	2	*-*	hypothetical protein	IE3
E2348C_2129	3	*pduF*	propanediol diffusion facilitator	
E2348C_2396	3	*ais*	hypothetical protein	
E2348C_2607	3	*cysA*	sulfate/thiosulfate transporter subunit	
E2348C_2608	3	*cysW*	sulfate transport system permease W protein; membrane component of ABC superfamily	
E2348C_2609	3	*cysU*	sulfate, thiosulfate transport system permease T protein; membrane component of ABC superfamily	
E2348C_2610	2	*cysP*	thiosulfate transporter subunit	
E2348C_2705	5	*-*	predicted glycosyl transferase	
E2348C_2915	39	*espC*	extracellular serine protease EspC	IE5
E2348C_2916	4	*-*	T3SS secreted effector EspG homolog	IE5
E2348C_2917	5	*-*	hypothetical protein	IE5
E2348C_2918	3	*-*	hypothetical protein	IE5
E2348C_2920	25	*-*	hypothetical protein	IE5
E2348C_3020	3	*cysC*	adenosine 5′-phosphosulfate kinase	
E2348C_3021	2	*cysN*	sulfate adenylyltransferase, subunit 1	
E2348C_3022	3	*cysD*	sulfate adenylyltransferase, subunit 2	
E2348C_3025	2	*cysH*	3′-phosphoadenosine 5′-phosphosulfate reductase	
E2348C_3026	3	*cysI*	sulfite reductase, β subunit, NAD(P)-binding, heme-binding	
E2348C_3027	3	*cysJ*	sulfite reductase, alpha subunit, flavoprotein	
E2348C_3262	2	*-*	predicted acyl-CoA synthase	
E2348C_3264	2	*-*	predicted acyl carrier protein	
E2348C_3839	9	*yiaY*	predicted Fe-containing alcohol dehydrogenase	
E2348C_3929	4	*yicJ*	predicted transporter	±LEE
E2348C_3930	7	*espF*	LEE-encoded effector EspF	LEE
E2348C_3931	100	*orf29*	component of T3SS, SsaH family	LEE
E2348C_3932	96	*escF*	T3SS structure protein EscF	LEE
E2348C_3933	73	*cesD2*	chaperone CesD2	LEE
E2348C_3934	20	*espB*	translocator EspB	LEE
E2348C_3935	66	*espD*	translocator EspD	LEE
E2348C_3936	97	*espA*	translocator EspA	LEE
E2348C_3937	100	*sepL*	secretion switching protein SepL	LEE
E2348C_3938	80	*escD*	T3SS structure protein EscD	LEE
E2348C_3939	100	*eae*	intimin Eae	LEE
E2348C_3940	62	*cesT*	chaperone CesT	LEE
E2348C_3941	39	*tir*	translocated intimin receptor Tir	LEE
E2348C_3942	73	*map*	LEE-encoded effector Map	LEE
E2348C_3943	79	*cesF*	chaperone CesF	LEE
E2348C_3944	38	*espH*	LEE-encoded effector EspH	LEE
E2348C_3945	54	*sepQ*	T3SS structure protein SepQ	LEE
E2348C_3946	37	*orf16*	hypothetical protein	LEE
E2348C_3947	25	*orf15*	hypothetical protein	LEE
E2348C_3948	14	*escN*	translocator EscN	LEE
E2348C_3949	39	*escV*	translocator EscV	LEE
E2348C_3950	27	*mpc*	regulator Mpc	LEE
E2348C_3951	15	*espZ*	LEE-encoded effector EspZ	LEE
E2348C_3952	39	*rorf8*	chaperone of T3SS Rorf8	LEE
E2348C_3953	47	*escJ*	T3SS structure protein EscJ	LEE
E2348C_3954	46	*sepD*	secretion switching protein SepD	LEE
E2348C_3955	46	*escC*	T3SS structure protein EscC	LEE
E2348C_3956	45	*cesD*	chaperone CesD	LEE
E2348C_3957	47	*grlA*	positive regulator GrlA	LEE
E2348C_3958	28	*grlR*	negative regulator GrlR	LEE
E2348C_3959	44	*rorf3*	hypothetical protein	LEE
E2348C_3960	13	*escU*	T3SS structure protein EscU	LEE
E2348C_3961	9	*escT*	T3SS structure protein EscT	LEE
E2348C_3962	9	*escS*	T3SS structure protein EscS	LEE
E2348C_3963	10	*escR*	T3SS structure protein EscR	LEE
E2348C_3964	9	*orf5*	component of T3SS	LEE
E2348C_3965	6	*orf4*	component of T3SS	LEE
E2348C_3966	7	*orf3*	component of T3SS	LEE
E2348C_3967	6	*orf2*	component of T3SS	LEE
E2348C_3968	26	*ler*	transcription regulator Ler	LEE
E2348C_3970	12	*espG*	LEE-encoded effector EspG	LEE
E2348C_3971	58	*rorf1*	hypothetical protein	LEE
E2348C_4442	4	*eptA*	predicted metal dependent hydrolase EptA	
pMAR2_097	3	*-*	putative glutamate racemase	pMAR2.

Fold activation shows expression in *ler* ^−+^/*ler* ^−^ cells. MGE, mobile genetic element; PP, prophage; IE, integrative element; LEE, locus of enterocyte effacement; ±LEE, directly adjacent to the LEE; pMAR2, plasmid. For each gene reported, *t*-test P-value was less than 0.05.

**Table 4:** EPEC genes repressed 2-fold or more by Ler at late-log phase (OD_600_ = 0.9).

Systematic Gene Name	Fold Repression	Common Gene Name	Gene Product	MGE
E2348C_0243	3	*yagU*	conserved inner membrane protein	±IE1b
E2348C_1068	2	*ycdO*	hypothetical protein	
E2348C_1662	2	*ydfI*	predicted mannonate dehydrogenase	
E2348C_1664	3	*rspB*	predicted oxidoreductase, Zn-dependent & NAD(P)-binding	
E2348C_1665	3	*rspA*	predicted dehydratase	
E2348C_3384	2	*uxaA*	altronate hydrolase	
E2348C_3385	2	*uxaC*	uronate isomerase	
E2348C_3401	3	*yhaO*	predicted transporter	
E2348C_3751	2	*hdeB*	acid-resistance protein	
E2348C_3752	2	*hdeA*	stress response protein acid-resistance protein	
E2348C_4130	3	*metE*	5-methyltetrahydropteroyltriglutamate-homocysteine S-methyltransferase	
E2348C_4624	9	*fimD*	outer membrane usher protein, type 1 fimbrial synthesis	

Fold repression shows expression in *ler* ^−^/*ler* ^+^ cells. ±IE1b, directly adjacent to IE1b. For each gene reported, *t*-test P-value was less than 0.05.

The strongest activation was generally observed for LEE genes, with the mostly high activated genes being *eae* at mid-log phase (58-fold) and *orf29* in late log phase (100-fold). Extra LEE genes with comparable levels of activation included *espC* (mid-log only), *pagP* and the gene encoding the T3SS secreted effector NleA. The maximum fold repression observed outside of the LEE was approximately 9-fold in mid-log phase (*fimD*) and approximately 6-fold in late log phase cells (*chuT*-*hmuV* heme utilization operon).

### Enterohaemorrhagic E. coli

In mid-log phase cells, only one gene passed the Benjamini and Hochberg MTC filter as being repressed (2-fold) by Ler. This was Z2974 on prophage CP-933T, encoding an unknown protein.

In late-log phase cells, 39 genes were found to be transcriptionally activated by Ler (2-fold or more; table 5). Thirty five of these genes are within the LEE (representing all major transcriptional units; activation between 4 and 32-fold). The remaining 4 extra-LEE activated genes encode: StcE (4-fold), EtpC (3-fold), SfpA (5-fold) and the putative cytochrome YhaI (36-fold). The *stcE* and *etpC* genes are located on plasmid pO157; SfpA is prophage-encoded and *yhaI* is not associated with a mobile genetic element.

**Table 5:** EHEC genes activated 2-fold or more by Ler at late-log phase (OD_600_ = 1.1).

Systematic Gene Name	Fold Activation	Common Gene Name	Gene Product	MGE
L7031	4	*stcE*	secreted zinc metalloprotease	pO157
L7032	2	*etpC*	component of type II secretion system for StcE	pO157
Z0955	5	*-*	systemic factor protein A homologue	PP (OI#36)
Z4458	36	*yhaI*	putative cytochrome	
Z5100	7	*espF*	LEE-encoded effector EspF	LEE (OI#148)
Z5102	24	*orf29*	component of T3SS, SsaH family	LEE (OI#148)
Z5103	14	*escF*	T3SS structure protein EscF	LEE (OI#148)
Z5104	12	*-*	chaperone CesD2	LEE (OI#148)
Z5105	9	*espB*	translocator EspB	LEE (OI#148)
Z5106	12	*espD*	translocator EspD	LEE (OI#148)
Z5107	24	*espA*	translocator EspA	LEE (OI#148)
Z5108	13	*sepL*	secretion switching protein SepL	LEE (OI#148)
Z5110	32	*eae*	intimin Eae	LEE (OI#148)
Z5111	30	*cesT*	chaperone CesT	LEE (OI#148)
Z5112	4	*tir*	translocated intimin receptor Tir	LEE (OI#148)
Z5113	12	*map*	LEE-encoded effector Map	LEE (OI#148)
Z5115	6	*espH*	LEE-encoded effector EspH	LEE (OI#148)
Z5116	5	*sepQ*	T3SS structure protein SepQ	LEE (OI#148)
Z5117	6	*orf16*	hypothetical protein	LEE (OI#148)
Z5118	6	*orf15*	hypothetical protein	LEE (OI#148)
Z5119	5	*escN*	translocator EscN	LEE (OI#148)
Z5120	6	*escV*	translocator EscV	LEE (OI#148)
Z5121	6	*mpc*	regulator Mpc	LEE (OI#148)
Z5122	6	*sepZ*	LEE encoded effector SepZ (EspZ)	LEE (OI#148)
Z5123	6	*rorf8*	chaperone Rorf8	LEE (OI#148)
Z5124	7	*escJ*	T3SS structure protein EscJ	LEE (OI#148)
Z5125	8	*sepD*	secretion switching protein SepD	LEE (OI#148)
Z5126	7	*escC*	T3SS structure protein EscC	LEE (OI#148)
Z5127	6	*cesD*	chaperone CesD	LEE (OI#148)
Z5128	8	*grlA*	positive regulator GrlA	LEE (OI#148)
Z5129	4	*grlR*	negative regulator GrlR	LEE (OI#148)
Z5134	8	*escS*	T3SS structure protein EscS	LEE (OI#148)
Z5135	9	*escR*	T3SS structure protein EscR	LEE (OI#148)
Z5136	7	*orf5*	hypothetical protein	LEE (OI#148)
Z5137	6	*orf4*	hypothetical protein	LEE (OI#148)
Z5138	6	*orf3*	hypothetical protein	LEE (OI#148)
Z5139	7	*orf2*	hypothetical protein	LEE (OI#148)
Z5140	27	*ler*	transcription regulator Ler	LEE (OI#148)
Z5142	5	*espG*	LEE-encoded effector EspG	LEE (OI#148)

Fold activation shows expression in *ler* ^−+^/*ler* ^−^ cells. ±IE1b, directly adjacent to IE1b. For each gene reported, *t*-test P-value was less than 0.05. MGE, mobile genetic element; pO157, plasmid; PP, prophage; OI#, O-island number. For each gene reported, t-test P-value was less than 0.05.

## Discussion

It is clear that in both the EPEC and EHEC strains of *E. coli* examined here, the LEE is the primary target for Ler activation: all major transcriptional units of the LEE are regulated by Ler, although the regulation of *LEE1* is growth phase dependent in EPEC, as noted below. Otherwise, in EPEC the Ler regulon is quite small, covering about 2% of the genome; in EHEC the regulon is even smaller and contains very few genes outside of the LEE. As the positive regulatory activity of Ler is known to be due to antagonism of H-NS repression (where studied) we would predict that all activated members of the Ler regulon are repressed by H-NS. However the H-NS regulon is very large and clearly not all H-NS repressed genes are activated by Ler [38]. An important question that therefore remains to be answered is: what provides specificity to Ler regulation? The specificity of action that we have observed (i.e. most of the strongly regulated genes are located within the LEE) is in agreement with the observations of Abe *et al.* relating to EHEC [36]. This specificity is consistent with Ler binding to a specific DNA structural motif, via an indirect readout mechanism, as suggested by an NMR analysis of Ler C-terminal domain-DNA complexes [39]. While some studies have shown evidence for specific binding of LEE promoters by Ler, the Chip-CHIP analysis of Abe *et al.* suggested that Ler was binding extensively (although not evenly) across the Sakai genome and the authors concluded that Ler has a low binding specificity [36].

Many but not all of the extra LEE members of the EPEC Ler regulon are located on mobile genetic elements (MGEs) and it is particularly striking that Ler negatively regulates a disproportionately high number of plasmid-borne genes, at least in mid-log phase EPEC: 9 genes from 3 different operons (10% of the total of 90 genes) on plasmid pMAR2 are shown to be regulated, while only 0.3% of the chromosomal genes (14 genes) are repressed. However by late log phase, no plasmid-borne genes are repressed by Ler. Similarly it is striking that 4 of the 5 extra-LEE genes found to be Ler-regulated in EHEC (likely members of the same operon) are located on a MGE (plasmid or prophage). In both bacteria the GC contents of chromosome is slightly higher than that of the plasmid: EDL933 and E2348/69 chromosomes are 50.4 and 50.6% respectively, while pO157 and pMAR2 are 47.6% and 48%. As genes located on MGEs with lower GC content are selectively silenced by H-NS [40], in evolutionary terms Ler activation could have been a useful means to “liberate” the expression of newly acquired pathogenicity factors.

**Figure 1:**
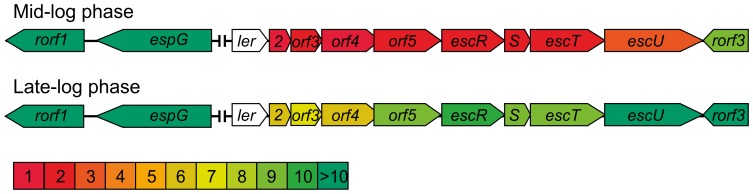
Growth phase dependent Ler regulation of the EPEC *LEE1* operon. While the other major operons of the EPEC LEE are regulated by Ler in a similar manner in both mid and late-log phase cultures, the *LEE1* operon (*ler* –*escU*) was strongly activated by Ler only in late log phase cultures. The *LEE1* operon and flanking genes are shown as block arrows and are coloured according to the fold activation seen in *ler* ^+^ cells. Fold activation values are not shown for the *ler* gene as this is partly deleted in *ler* ^−^ cells. The intergenic region between *ler* and *espG* has been contracted for clarity.

Across the genome, the Ler regulon is notably growth phase dependent in EPEC: in mid log phase (OD_600_ = 0.4) 27 extra-LEE genes are activated by Ler, while in late log phase (OD_600_ = 0.9) the number of activated extra-LEE genes is 43. In EPEC the regulation of the *LEE1* operon, but not the other operons of the LEE, differs between mid-log and late-log growth phases: at late log phase, all 41 genes within the LEE are strongly activated by Ler, along with the flanking predicted sugar transporter gene *yicJ*, while at mid-log phase the 7 genes in the *LEE1* operon before *escU* are not strongly (>2-fold) regulated (Figure 1; note that we do not comment on the regulation of the *ler* gene itself as the coding sequence is partly deleted in the mutant). While it is possible that we have introduced some artefactual corruption of *LEE1* regulation during mutation of *ler*, the observed activation of late-log phase cells suggests that there is no gross defect in the Ler regulatory circuit. This result indicates that the regulation of the *LEE1* promoter is somewhat different to that of other LEE promoters, possibly due to a complex balance between Ler autoregulation and activation. It is noteworthy that, while previous reporter gene analysis of the *LEE1* promoter has indicated that it is autorepressed by Ler, our results indicate that it may be activated, a difference that may reflect the growth phase dependence of the effects observed here [15]. No corresponding differential regulation of the *LEE1* operon was observed in late log phase EHEC; in the mid-log phase cultures none of the LEE genes passed the MTC filter, but if the filter is not applied then *LEE1* seems to be similarly regulated in mid-log and late-log phase cultures. While Sperandio *et al.* found that the *LEE4* operon (*sepL*-*espF*) was constitutively expressed at a high level in EHEC and insensitive to Ler regulation [20], we have found it to be clearly Ler-dependent in both EHEC and EPEC strains. The observed difference could have resulted from selection of a promoter fragment for reporter gene assays that lacks the full complement of H-NS binding sites.

There are a number of Ler-activated genes in the EPEC regulon that are outside of the LEE but may be involved in pathogenicity. As noted above, *espC* is already known to be *ler*-regulated and is one of the mostly highly (22-fold) activated genes in mid-log phase cells. PagP, the palmitoyl transferase for lipid A is strongly regulated at both mid and late log phases (9-fold and 15-fold respectively). Palmitoylated lipid A supposedly protects bacteria from host immune defences (e.g. CAMPs) and attenuates their activation through the TLR4 signal transduction pathway [41]. E2348C_0684, strongly regulated along with its downstream neighbour, encodes a SfpA (systemic factor protein A)-like protein: SfpA is a porin involved in systemic disease in *Yersinia enterocolitica* [42]. A homologue of *sfpA* (ECs0814) in the Sakai strain of EHEC was previously observed to be Ler regulated [36]. The *rcsA* gene, which encodes a positive regulator of the serotype-specific group I K (capsular) antigen is activated by Ler in late log phase, although not at the earlier growth point [43]. This may reflect an impact of capsule production on the intimate attachment of EPEC bacteria to the gut epithelium, however, no regulation of the *wza* promoter (target for RcsA in *E. coli* K-12) was apparent. It is worth noting at this point that a *ler* mutant of EPEC was previously found to be defective for colonisation of *Caenorhabditis elegans* [44]. This requirement for Ler was found to be independent of T3SS encoded by the LEE. This effect is presumably due to one or more of these extra-LEE members of the Ler regulon which are essential pathogenicity factors in a *C. elegans* infection but are not involved in T3S (and are not effectors delivered by the T3SS). Several non-LEE encoded effector genes, whose products are secreted via the T3SS, are Ler-regulated in EPEC, including the operon of five genes from *nleI/G* to *nleF* (Ler regulation of a homologue of *nleA* is already known to occur in EHEC [33]) and a homolog of the *espG* gene located next to the *espC* gene which is also Ler regulated (see above). There is also clear evidence for the transcriptional regulation of *nleH* and *espJ* homologues at late log phase. While it may be unsurprising that effectors secreted via the T3SS are coregulated with the LEE, previous studies in EHEC and *C. rodentium* have not found these two genes to be regulated by Ler [34], [45]. Only 4 extra-LEE genes were identified as part of the EHEC Ler regulon: *sfpA*, as discussed above; *stcE*, encoding a protease that is known to be involved in intimate adherence and inhibition of complement-mediated lysis [32], [46]; *etpC*, located immediately downsteam of *stcE* and encoding a component of the pO157-encoded type II secretion system for StcE is also known to be involved in adherence and intestinal colonization [47] and the putative cytochrome gene *yhaI*. Assuming that *etpC* is in the same operon as *stcE*, only the last of these is a novel observation.

We have also identified a number of EPEC genes that are repressed by Ler, including the “plasmid-encoded regulator” operon *perABC*, located on the EPEC adherence factor (EAF) plasmid pMAR2 [48]. PerA protein activates transcription of the *bfp* operon, encoding bundle-forming pili [49]. These pili are involved in formation of an initial attachment between EPEC cells and the gut epithelium that occurs prior to AE lesion formation, therefore down-regulation of *bfp* expression with LEE expression is consistent with the known program of infection [50]. PerC protein is known to activate *ler* [17], [18], [51] and therefore this result suggests the existence of a negative feedback loop, previously undescribed, that ultimately autoregulates expression of Ler (and therefore the LEE) and may be involved in a down-regulation of *ler* transcription after the initial stages of infection [52]. Regulation of the *per* operon by Ler, the gene for which is known to be regulated by quorum sensing (QS), would account for the previously observed “indirect” QS regulation of *perA* [20]. The repressive effect of Ler on *perA* presumably also explains the up-regulation of the bundle-forming pili (*bfp*) operon in the *ler* knockout mutant. Neither of these phenomena (which were only observed in mid-log phase cells) have so far been reported in the literature, although Elliot *et al.* reported Ler regulation of non-BFP fimbriae, while Leverton and Kaper described an inverse relationship between expression of *ler* and *bfpA* in the presence of HEp-2 cells [12], [52]. Ler repression of acid resistance genes – previously noted by Abe *et al.* in the Sakai strain [36] - may reflect an accessory mechanism to assist in tight regulation of these genes, preventing inappropriate expression in the lower regions of the GI tract where acid resistance is not required.

Overall the data reported here suggests that the Ler regulon for enteropathogenic and enterohaemorrhagic strains of *E. coli* is mainly focused on the type III secretion system genes in the LEE, but also includes unlinked pathogenicity genes. The regulon is growth phase dependent and, at least in strain E2348/69, is composed of both positively and negatively regulated genes. Additionally, in enteropathogenic *E. coli*, the observed negative regulation by Ler of PerC, itself a positive regulator of the *ler* promoter, suggests the existence of a negative feedback loop involving these two proteins.

## Materials and Methods

### Bacterial strains and plasmids

Bacterial strains used or constructed during this study are detailed in table 6 and plasmids used or constructed are detailed in table 7. Standard techniques for recombinant DNA manipulations were used throughout this work. All cloned sequences were checked using the University of Birmingham Functional Genomics Facility (http://www.genomics.bham.ac.uk/sequencing.htm).

**Table 6:** Bacterial strains used or constructed in this study.

Strain	Description	Reference
*E. coli* EDL933 TUV 93-0	Enterohaemorrhagic *E. coli* Stx- derivative	Arthur Donohue- Rolfe, Tufts Cummings School of Veterinary Medicine; [59]
*E. coli* E2348/69	Enteropathogenic *E. coli*	[60]
LBEC1	EDL933 Δ*ler*	This study
LBEC2	E2348/69 Δ*ler*	This study

**Table 7:** Plasmids used or constructed in this study.

Plasmid	Description	Reference
pACBSCE	Genedoctoring λRed/*Sce*I suicide expression plasmid	[55]
pDOC-C	Genedoctoring donor plasmid	[55]
pLBP100	EHEC Δ*ler*-*aphA* cassette donor plasmid (pDOC-C derivative)	This study
pLBP101	E2348/69 Δ*ler*- *aphA* cassette donor plasmid (pDOC-C derivative)	This study
pCP20	Temperature sensitive FLP expression plasmid	[56]
pJW15Δ1-100	*melRp* expression plasmid	[52]
pSI04	*melRp*-*ler* expression plasmid (pJW15Δ1-100 derivative)	This study

### Construction and validation of *E. coli* mutant strains and *ler* expression plasmid

The *ler* expression plasmid pSI04 was derived from pJW15Δ1-100 [53] by cloning EHEC *ler* CDS as a *Nsi*I-*Hin*dIII fragment under the control of the *melR* promoter and SD site [54].

Non-polar *ler* knockout mutants of *E. coli* strains EDL933 and E2348/69 were constructed using a λRed based method (GeneDoctoring) [55] to replace the majority of the *ler* gene with a kanamycin resistance cassette. Recombination cassettes designed to replace the central portion of *ler* with a kanamycin resistance gene (*aphA*) were amplified from a pDOC-K template using a conserved forwards primer (LER-KO-F: taatagcttaaaatattaaagcATGCGGAGATTATTTATTATGAATATGG-TGGCTGGAGCTGCTTCGAA) in combination with strain specific reverse primers (LER-KO-EHEC-R: catttaattatttcatgTTAAATATTTTTCAGCGGTATTATTTCTTCT-CTCGAGATATGAATATCCTCCTTAG and LER-KO-EPEC-R: catttaattattttatgTTAAATATTTTTCAGCGGTATTATTTCTTCT-CTCGAGATATGAATATCCTCCTTAG) and a proofreading DNA polymerase (Velocity, Bioline). The blunt-ended cassettes were ligated into *Eco*RV-digested donor plasmid pDOC-C to generate donor plasmids carrying EHEC and EPEC specific *ler*− *aphA*+ knockout cassettes. These plasmids were used together with pACBSCE to replace the Ler coding sequence with the kanamycin resistance gene cassette. The antibiotic resistance cassette was subsequently removed via flanking Flp recombination target (FRT) sites using the temperature sensitive FLP expression plasmid pCP20 [56]. The Δ*ler* locus in the resulting unmarked mutants encoded the first and last 9 aa of Ler (first 3 aa of the shorter Ler protein as described by Mellies *et al.* [57]) sandwiching a central “scar region” derived from the FLP recombinase sites encoding 29 (non-Ler) amino acids. Loss of all three plasmids (pDOC-derived donor plasmids and pACBSCE, pCP20) involved in mutagenesis was confirmed by antibiotic resistance profiling. The DNA sequence surrounding the recombination site was checked by sequencing across the knockout locus from primers designed to bind flanking sites. Recombinant strains were designated LBEC1 (EDL933 Δ*ler*) and LBEC2 (E2348/69 Δ*ler*).

The absence of gross unwanted deletions in the mutant strains was confirmed by comparative genomic hybridization (CGH) of labeled genomic DNA extracted from wild-type and mutant (Δ*ler*) strains. No missing loci, other than the desired deletion of *ler*, were apparent. Growth curves were assessed for LBEC1 and LBEC2 strains in comparison to parental wild-types and no gross defects in growth were observed (a small growth advantage consistent with predicted increased fitness due to reduced expression of T3SS was sometimes observed for the mutant strains on growth in inducing Dulbecco's Modified Eagle Medium (DMEM) medium, but this was neither statistically significant nor reproducible).

The *ler* mutation in strains LBEC1 and LBEC2 was successfully complemented using the *ler* expression plasmid pSI04 resulting in the restoration of a functional T3SS, as confirmed by the fluorescent actin staining (FAS) test (i.e. via microscopic assessment of AE lesion formation (table 8) [58]. Subconfluent HeLa cell monolayers on glass coverslips were infected for 4 hours at 37°C with a 1∶100 dilution of an overnight LB broth culture of *E. coli* diluted in DMEM buffered with 25 mM HEPES. Following fixation in 4% formalin for 20 minutes and permeabilization in 0.1% Triton in PBS for 4 minutes, cells were stained with 12 µg/ml FITC conjugated phalloidin (Sigma) for 20 minutes at room temperature [54]. Bacterial cells were simultaneously stained with 10 µg/ml propidium iodide (Invitrogen).

**Table 8:** Summary of FAS^1^ testing of complementation of *E. coli ler* mutant strains.

Strain	Plasmids	*ler*	Adherence	FAS
EDL933	-	WT	++	++
LBEC1	pJW15Δ100	Δ*ler*	++	−
LBEC1	pSI04	Δ*ler* (*+ ler in trans*)	++	++
E2346/69	-	WT	++++	++++
LBEC2	pJW15Δ100	Δ*ler*	++++	−
LBEC2	pSI04	Δ*ler* (*+ ler in trans*)	++++	++++

^1^ FAS, fluorescent actin staining test.

### RNA Purification

Quadruplicate overnight cultures of WT and Δ*ler* strains, grown in LB broth (Miller formulation) were diluted 1/100 into DMEM buffered with 25 mM HEPES and incubated at 37°C, with aeration by shaking at 200 rpm (i.e. inducing conditions for expression of the LEE). Samples were harvested at mid and late log phases of growth (OD_600_ of 0.4 and 0.9 for EPEC; 0.5 and 1.1 for EHEC). Messenger RNA was stabilized immediately by pipetting the samples directly into RNAprotect Bacteria reagent (Qiagen) before purification of total RNA using the RNeasy Mini Kit with on-column DNase digestion (Qiagen).

### Microarray labelling and hybridization

The concentration of RNA was determined using a spectrophotometer (ND-1000; NanoDrop). Five hundred nanograms of total RNA was used for labelling, and aRNA was synthesized with the Ambion MessageAmp™ II-Bacteria RNA Amplification Kit according to the recommendations of the manufacturer and labeled with the Cy3 or Cy5 monoreactive dye pack (GE Healthcare). Labeled aRNA was purified with Qiagen RNeasy MinElute clean up kit according to the manufacturer's instructions and quantified using a spectrophotometer (ND-1000; NanoDrop). The 8×15,000 (15K) DNA high-density microarrays of E2348/69 and EDL933 were designed by Oxford Gene Technology (Oxford OX5 1PF, United Kingdom) and validated by the University of Birmingham *E. coli* Centre (UBEC) (United Kingdom). During validation, three 60-mer probes per predicted gene were designed for all the open reading frames (ORFs) in the chromosome and plasmids of each one of the two *E. coli* strains used in this study. For each of the designed probes, a mismatch probe (containing 3 mismatches per 60-mer probe at positions 10, 25, and 40) was also generated. These mismatch probes and the perfect-match probes designed against each strain were placed on an array (4×44k) in triplicate. This array was hybridized with genomic DNA and a pool of mRNA representing conditions in which as many genes as practicable would be induced (derived from an equimolar pool of total RNA from *E. coli* grown in morpholinepropanesulfonic acid (MOPS) minimal medium at 30°C mid-log phase, 37°C for mid-log phase, and 37°C for stationary phase). The results were processed to select the best-performing probe for each gene. This derived and optimized probe set was printed in a random pattern in triplicate by Agilent Technologies on an 8×15K array for each strain and used in this study. For each of the four biological replicates equal quantities (300 ng) of Cy5- and Cy3-labeled aRNA were added to hybridization solution, and hybridization was performed using the Gene Expression hybridization kit (Agilent Technologies).

### Analysis of Microarray Data

The microarray images were analyzed using GenePix software v6 (Axon Instruments). The data were imported into GeneSpring, version 7 (Agilent). A Lowess curve (locally weighted linear regression curve) was fitted to the plot of log intensity versus log ratio, and 40% of the data were used to calculate the Lowess fit at each point. The curve was used to adjust the control value for each measurement. If the control channel signal was below a threshold value of 10, then 10 was used instead.

For each strain data set a list of genes was prepared showing at least 2-fold differential expression levels between the *ler* and wild type samples for each one of the two growth conditions by using Student's *t*-test and applying the Benjamini and Hochberg false discovery rate (multiple testing correction, MTC) test with a *p* value cut off of 0.05.
